# Case Report: Molecular Analyses of Cell-Cycle-Related Genes in Cortical Brain Tissue of a Patient with Rasmussen Encephalitis

**DOI:** 10.3390/ijms25158487

**Published:** 2024-08-03

**Authors:** João Ismael Budelon Gonçalves, Vinicius Rosa de Castro, William Alves Martins, Fernando Antonio Costa Xavier, Jaderson Costa Da Costa, Eliseu Paglioli Neto, André Palmini, Daniel Rodrigo Marinowic

**Affiliations:** 1 Brain Institute of Rio Grande do Sul, Porto Alegre 90610-000, Brazil; joao.goncalves@edu.pucrs.br (J.I.B.G.); fxavier@pucrs.br (F.A.C.X.); jcc@pucrs.br (J.C.D.C.); 2 Graduate Program in Medicine and Health Sciences, Medical School, Pontifical Catholic University of Rio Grande do Sul, Porto Alegre 90619-900, Brazil; vinicrc@hotmail.com; 3 Epilepsy Surgery Program, Saint Lucas Hospital, Pontifical Catholic University of Rio Grande do Sul, Porto Alegre 90610-001, Brazil; william.martins@edu.pucrs.br (W.A.M.); epaglioli@hotmail.com (E.P.N.); apalmini@uol.com.br (A.P.)

**Keywords:** Rasmussen’s encephalitis, Rasmussen, cell cycle, inflammatory, encephalopathy, BDNF, brain-derived neurotrophic factor, qPCR, array, cytokines

## Abstract

Rasmussen’s encephalitis (RE) stands as a rare neurological disorder marked by progressive cerebral hemiatrophy and epilepsy resistant to medical treatment. Despite extensive study, the primary cause of RE remains elusive, while its histopathological features encompass cortical inflammation, neuronal degeneration, and gliosis. The underlying molecular mechanisms driving disease progression remain largely unexplored. In this case study, we present a patient with RE who underwent hemispherotomy and has remained seizure-free for over six months, experiencing gradual motor improvement. Furthermore, we conducted molecular analysis on the excised brain tissue, unveiling a decrease in the expression of cell-cycle-associated genes coupled with elevated levels of BDNF and TNF-α proteins. These findings suggest the potential involvement of cell cycle regulators in the progression of RE.

## 1. Introduction

Rasmussen’s encephalitis (RE) is a rare chronic inflammatory encephalopathy. The clinical profile encompasses severe refractory epilepsy, hemiplegia, impairments in motor skills and speech, dementia, and the encephalitis condition marked by brain inflammation leading to progressive atrophy of one cerebral hemisphere [1]. The annual incidence per 10 million people was described to be 2.4 in Germany and 1.7 in the UK. Currently, the 2005 European consensus reported by Bien is still the accepted guideline for the pathogenesis, diagnosis, and treatment of RE [2].

The primary cause of RE remains unknown; histopathological hallmarks include cortical inflammation, neuronal loss, and gliosis localized to one cerebral hemisphere, whereas the involvement of T lymphocytes has also been described [3,4]. At this moment, surgery is the only potential cure. Early diagnosis of RE is imperative to initiate interventions aimed at arresting disease progression and ameliorating patient outcomes [1,5]. Therefore, a deeper understanding of the molecular mechanisms of the disease is needed to develop non-invasive treatments and novel biomarkers. 

Here, we report a patient with RE who underwent hemispherotomy and remains seizure-free after more than 6 months after surgery, with progressive motor improvement. Further, we performed molecular analysis of resected brain tissue and found a down-regulation of cell-cycle-related genes, possibly due to an increase in BDNF protein levels.

## 2. Case Report

A 13-year-old boy who initially presented a bilateral tonic-clonic seizure. A few weeks prior, he had a self-limited viral illness, and his medical history and development had been otherwise uneventful. The onset of symptoms began approximately 2 years earlier with spasms in the limbs on the left, eventually associated with generalized tonic-clonic seizures. In addition, there was cephalic and ocular rotation to the right and hypertonia of the limbs on the left lasting an average of 5 min. He was started on valproate but, a few months later, developed difficult-to-control seizures with eye and head version to the left and left hypertonia. Later on, he presented focal myoclonic jerks in his left arm several times a day, at times progressing to his left leg and face. 

**Figure 1:**
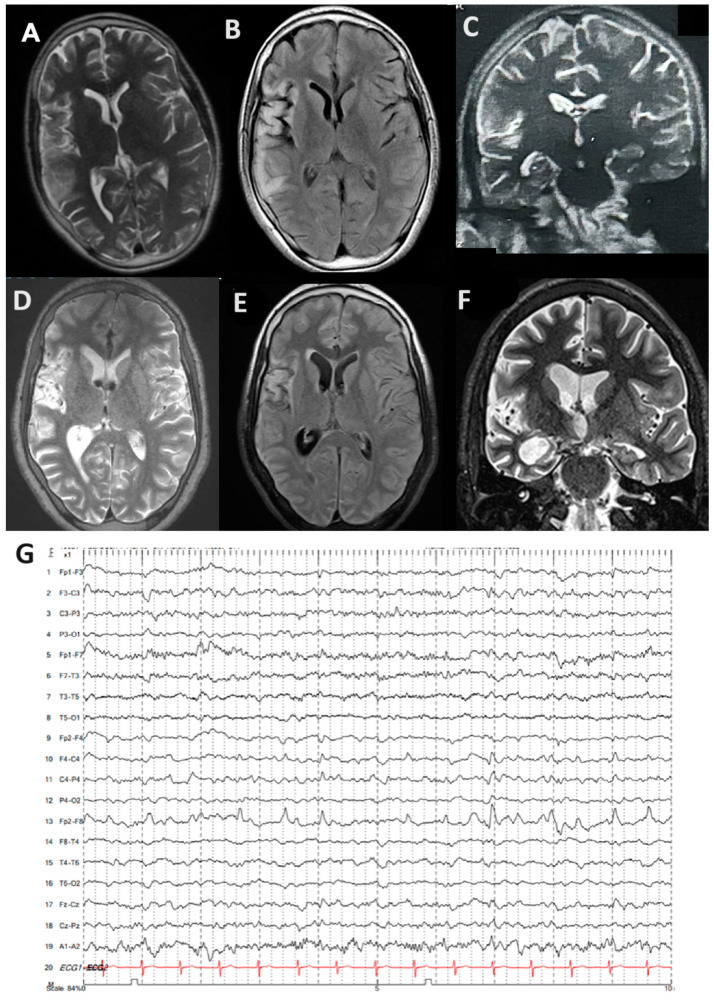
(**A**–**G**) MRI and EEG from patient with RE: (**A**–**C**) brain MR images highlighting T2 and T2 FLAIR. Hyperintensity in the right frontotemporal-insular region suggests subacute encephalitis with some degree of cortical atrophy; (**D**–**F**) brain MRI three months later shows extensive right hemisphere atrophy, predominating in the perisylvian region and affecting the caudate; (**G**) representative EEG with continuous rhythmic activity detected in the Fp2-F8 region.

Carbamazepine and lacosamide were added on with no improvement, and he developed progressive weakness in his left arm and leg. There was no family history of epilepsy, viral serologies (herpes simplex virus, EBV, and CMV), and autoimmunity markers (ANA and RF). He was then referred to our tertiary epilepsy center, at which time neurological examination showed spastic left hemiparesis and moderate-to-severe dysarthria, with continuous left hemiclonic motor seizures (epilepsia partialis continua [EPC]). By arrival, his MRI showed progressive right hemisphere and caudate atrophy compared to his previous MRI, as well as hyperintensities in the right temporal and frontal lobes, extending throughout the right insula (Figure 1A–F). EEG also showed slowing in the right hemisphere with periodic sharp waves over the right frontotemporal and parietal regions, as well as frequent electrographic seizures (Figure 1G).

Despite all measures and antiseizure drugs, he was refractory to treatment and had been admitted to the pediatric ICU for uncontrolled seizures and status epilepticus. This constellation of symptoms led us to diagnose probable Rasmussen’s encephalitis (RE), and he underwent a right functional hemispherotomy. On the first post-operative day, he had no more seizures and was awake and responsive; however, he developed a transitory right third nerve palsy. He has remained seizure-free for the last six months post-surgery (Engel IA) and has shown progressive motor improvement of the left leg and arm. This set of findings allowed us to consider a diagnosis of Rasmussen’s encephalitis. A cortical tissue sample obtained after functional hemispherotomy and histopathology confirmed RE. Excised tissue was subjected to molecular analysis to evaluate the expression of cell-cycle-related genes, as well as protein levels of inflammatory cytokines and neurotrophic factors.

## 3. Results

### 3.1. Cortex Sample Characterization

All patients involved had refractory epilepsy and were referred to the Epilepsy Surgery Program at Hospital São Lucas da PUCRS for surgery due to conditions of RE and temporal lobe epilepsy with hippocampal sclerosis (TLE-HS; control group). The cortex samples of the control group were obtained via residual resection of TLE-HS (*n* = 5). The decision to perform an anterior temporal lobectomy instead of an amygdalohippocampectomy was made by the program’s medical team without the participation of the researchers. No additional cortex fragments were removed during surgery. The histological sections were bright grayish white with heterogeneous areas in the transition between white and gray matter of two turns. The brain parenchyma showed areas of reactive gliosis, zones of edema, neuronal loss in the subpial region, and foci of lymphocytic infiltrate without atypia. The set of histopathological findings associated with clinical data is compatible with RE.

### 3.2. PCR Array for Cell-Cycle Related Genes

To analyze the expression of genes associated with cell cycle regulation, we employed the RT^2^ Profiler™ PCR Array Human Cell Cycle (Qiagen, Germantown, MD, USA), which allows for the simultaneous evaluation of up to 88 key genes involved in this process. Total RNA was isolated from the samples using TRIzol™ reagent (Invitrogen, Pittsburgh, PA, USA) following the manufacturer’s protocol. cDNA was synthesized from 1 μg of RNA using the RT^2^ First Strand Kit (Qiagen, USA) according to the manufacturer’s instructions. The RT-qPCR array was performed using the QuantStudio™ 3 Real-Time PCR System (Thermo Fisher Scientific, Waltham, MA, USA) with cycling conditions as specified in the RT^2^ Profiler™ PCR Array’s instructions. The data were analyzed using the ΔΔCt method, normalizing the expression levels to the housekeeping genes provided in the array. The results were expressed as fold changes relative to the control group.

**Figure 2:**
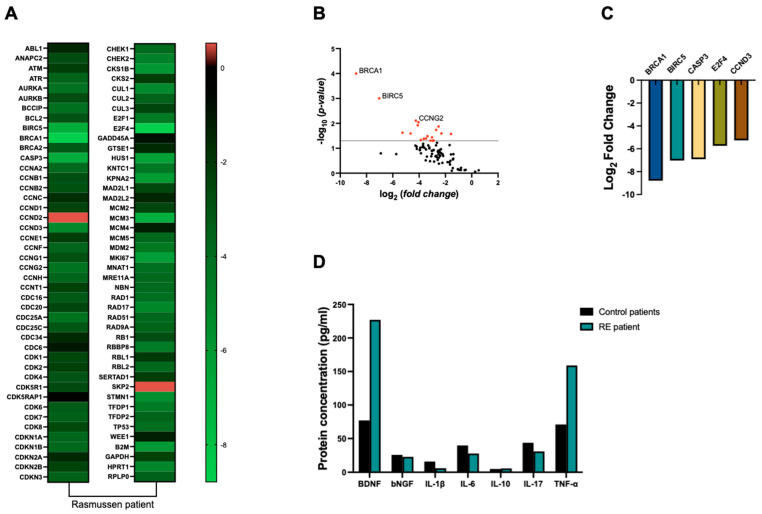
Molecular analysis of brain tissue of patient with RE. (**A**) Heatmap from RT 2 Profiler™ PCR Array human cell cycle pathway genes. Data are representative of log2 fold change difference from a control group (*n* = 5) and a Rasmussen patient. (**B**) Volcano plot for control group versus Rasmussen patient showing significantly down-regulated genes which passed the −1.3 (*p* < 0.05) threshold for log2 fold change difference. *p*-values are calculated based on a Student’s *t*-test of the mean 2 −ΔCT values for each gene in the control group versus the Rasmussen patient. (**C**) Log2 fold change from top five down-regulated genes. (**D**) Protein levels from Luminex immunoassay.

Our findings revealed an overall down-regulation of cell-cycle-related genes, particularly *Breast cancer type 1 susceptibility protein* (BRCA1) (8-fold), *baculoviral IAP repeat containing 5* (BIRC5) (7-fold), *caspase 3* (CASP3) (6.9-fold), *E2F transcription factor 4* (E2F4) (5.7-fold), and *cyclin D3* (CCDN3) (5.2-fold), as depicted in Figure 2A–C, when compared to control samples.

### 3.3. Cytokines and Neurotrophic Factors Protein Levels

As inflammation is considered a hallmark of RE, we decided to analyze the concentration of inflammatory cytokines in the resected brain tissue. 

The serum levels of brain-derived neurotrophic factor (BDNF), beta-nerve growth factor (bNGF), anti-tumor necrosis factor-α (TNF-α), interleukin-1 beta (IL-1β), interleukin-6 (IL-6), interleukin-10 (IL-10), and interleukin-17 (IL-17) were quantified using a multiplex bead-based Luminex assay (MilliporeSigma, Burlington, MA, USA). The assay was conducted following the manufacturer’s instructions. Standards, controls, and reagents were prepared accordingly, and serum samples were thawed on ice and diluted in assay buffer. A 96-well microplate was pre-wetted, and 50 µL of standard, control, or diluted sample was added to each well, followed by 50 µL of antibody-immobilized beads. The plate was incubated on a shaker for 2 h at room temperature in the dark NS washed three times, and then 50 µL of biotinylated detection antibody was added. After a 1 h incubation, the plate was washed again, and 50 µL of streptavidin–phycoerythrin was added. Following a final 30 min incubation and wash, the beads were resuspended in wash buffer and analyzed using a Luminex MAGPIX instrument with data acquisition through xPONENT 4.3 software.

Our analysis revealed a 2-fold increase in TNF-α levels in RE tissue compared to control samples. However, no significant differences were observed in the concentrations of other cytokines, including IL-1β, IL-6, IL-10, and IL-17, between RE and control groups (Figure 2D). Our findings revealed a substantial 2.9-fold increase in BDNF protein levels in RE tissue compared to control samples. Conversely, NGF levels remained relatively consistent between RE and control groups (Figure 2D).

## 4. Discussion

RE is a rare epileptic disorder, typically emerging during childhood, marked by a gradual unilateral hemispheric degeneration of the brain [2]. While the exact etiology of RE remains elusive, it is increasingly recognized as an autoimmune-mediated disorder, leading to investigations into treatments targeting the immune response [6]. In contrast to many cell types, neurons are thought to lose their ability to divide once they have matured, remaining predominantly quiescent within the adult nervous system. Yet, reactivation of the cell cycle in adult neurons is an initial indicator of neurodegeneration and CNS injury [7]. Therefore, we decided to evaluate THE expression of cell-cycle-related genes in the brain tissue of a patient with RE.

In this work, we found a general decreased expression of cell-cycle-related genes in brain tissue from the patient with RE, where the genes BRCA1, BIRC5, CASP3, E2F4, and CCDND3 were more than 5-fold down-regulated.

The BRCA1 gene plays a vital role in DNA repair and cellular responses to DNA damage, with associations to senescence and various neurological disorders [8,9]. Negative regulation of BRCA1 expression in the brain, as seen in the patient with RE, may impact neural tissue homeostasis, altering responses to medication and tissue excitability thresholds. Knockdown mouse models of BRCA1 show reduced cell size and dendritic branching, alongside impaired long-term potentiation, indicating BRCA role in synaptic plasticity crucial for learning and memory [10].

Several studies suggest that neuronal death in conditions such as ischemia, seizures, and brain diseases involve programmed cell death, including apoptosis [11]. Cyclin Ds, crucial for mitotic control, serve as markers by which to assess neuronal progression through the cell cycle under pathology [12]. Brain tissue from the patient with RE showed significantly reduced expression of CCND3 and E2F4 genes compared to controls. The negative expression of CCND3 in the cortical tissue of the patient with RE may be an important factor in the instability of the damaged tissue and the progression of seizures and refractoriness because the expression of cell cycle regulators in healthy differentiated neurons is not related to neuronal proliferation but rather to a role possibly linked to neuronal plasticity and stability.

E2F4, a transcriptional repressor, plays a vital role in cell cycle arrest and is crucial for the proliferation and survival of mouse embryonic stem cells, decreasing histone acetylation at cell cycle gene promoters [13]. In RE, brain tissue may undergo aberrant reorganization after seizures due to molecular deficiencies in DNA repair and cell cycle control, potentially leading to improper cell fate determination.

Another gene down-regulated in RE tissue is BIRC5, which belongs to the inhibitor of apoptosis (IAP) gene family, which encodes proteins that prevent cell death via apoptosis. Evidence suggests that the IAP family is associated with regulating the progression of the intrinsic pathway during seizure-induced neuronal death [14]. Therefore, the reduction in BIRC5 expression may be a consequence of the patient’s successive seizures due to refractoriness. Marinowic et al. (2020) demonstrated through an induced pluripotent stem cell (iPSC) model from patients with focal cortical dysplasia (FCD) type 2b that the expression of CIAP1 was 20-fold decreased when compared to control brain tissue [15].

Caspase-3 is a protein involved in apoptosis, found to be significantly elevated in the temporal cortex of epilepsy patients compared to controls. While a caspase-3 inhibitor did not reverse neurodegeneration, it adversely affected axonal and dendritic integrity [16]. Cortical tissue from the patient with RE presented a negative fold change in caspase-3 expression, where a possible decrease in apoptosis mediated by this negative regulation may not have an anti-epileptogenic effect but instead may impair the structure and integrity of neurons following a seizure.

Notably, we also observed changes in the expression of the MCM3 gene, which is involved in genome replication, cell proliferation, and neuronal cell cycle activation [17]. The MCM3 protein is a component of the mini-chromosome maintenance (MCM) complex, which is essential for the initiation and elongation of DNA replication [17,18]. Altered expression of MCM3 can lead to dysregulation of the cell cycle and may contribute to the abnormal neuronal proliferation observed in Rasmussen’s encephalitis. This finding suggests that MCM3, along with other cell-cycle-related genes, might be involved in the pathophysiology of the disease, potentially through mechanisms that disrupt normal neuronal function and survival.

BDNF is a protein that belongs to the neurotrophin family which plays a crucial role in the survival, growth, and maintenance of neurons in the brain [19]. Here, we report that BDNF protein levels are increased in RE brain tissue. In the context of RE, an initial insult could lead to an increase in BDNF as a neuroprotective response. When excessive, BDNF inhibits cell cycle regulators, including apoptosis-related genes, thus promoting survival of damaged neurons [20]. However, these damaged neurons then stimulate the production of more BDNF, establishing a feedback loop. This loop amplifies neurodegeneration, ultimately worsening the condition.

Surgery remains the sole solution for addressing seizures induced by RE, with postoperative seizure-free rates ranging from 70% to 80%. After a failure to control the patient’s symptoms using medication, we decided to proceed to a functional hemispherotomy. Since surgery was performed, the patient has been free from seizures for the past six months according to the Engel IA classification. Additionally, he has displayed gradual motor enhancement in the left leg and arm.

## Data Availability

All data from this article are available from the corresponding author under reasonable request.
